# Prognostic estimation for acute ischemic stroke patients undergoing mechanical thrombectomy within an extended therapeutic window using an interpretable machine learning model

**DOI:** 10.3389/fninf.2023.1273827

**Published:** 2023-10-13

**Authors:** Lin Tong, Yun Sun, Yueqi Zhu, Hui Luo, Wan Wan, Ying Wu

**Affiliations:** ^1^Department of Radiology Intervention, Shanghai Putuo District Liqun Hospital, Shanghai, China; ^2^Department of Emergency, Shanghai Putuo District Liqun Hospital, Shanghai, China; ^3^Institute of Diagnostic and Interventional Radiology, Shanghai Jiao Tong University Affiliated Sixth People’s Hospital, Shanghai, China

**Keywords:** acute ischemic stroke, mechanical thrombectomy, extended therapeutic window, machine learning, prognosis prediction, Shapley additive explanation

## Abstract

**Background:**

Mechanical thrombectomy (MT) is effective for acute ischemic stroke with large vessel occlusion (AIS-LVO) within an extended therapeutic window. However, successful reperfusion does not guarantee positive prognosis, with around 40–50% of cases yielding favorable outcomes. Preoperative prediction of patient outcomes is essential to identify those who may benefit from MT. Although machine learning (ML) has shown promise in handling variables with non-linear relationships in prediction models, its “black box” nature and the absence of ML models for extended-window MT prognosis remain limitations.

**Objective:**

This study aimed to establish and select the optimal model for predicting extended-window MT outcomes, with the Shapley additive explanation (SHAP) approach used to enhance the interpretability of the selected model.

**Methods:**

A retrospective analysis was conducted on 260 AIS-LVO patients undergoing extended-window MT. Selected patients were allocated into training and test sets at a 3:1 ratio following inclusion and exclusion criteria. Four ML classifiers and one logistic regression (Logit) model were constructed using pre-treatment variables from the training set. The optimal model was selected through comparative validation, with key features interpreted using the SHAP approach. The effectiveness of the chosen model was further evaluated using the test set.

**Results:**

Of the 212 selected patients, 159 comprised the training and 53 the test sets. Extreme gradient boosting (XGBoost) showed the highest discrimination with an area under the curve (AUC) of 0.93 during validation, and maintained an AUC of 0.77 during testing. SHAP analysis identified ischemic core volume, baseline NHISS score, ischemic penumbra volume, ASPECTS, and patient age as the top five determinants of outcome prediction.

**Conclusion:**

XGBoost emerged as the most effective for predicting the prognosis of AIS-LVO patients undergoing MT within the extended therapeutic window. SHAP interpretation improved its clinical confidence, paving the way for ML in clinical decision-making.

## Introduction

Mechanical Thrombectomy (MT) has established its efficacy as a primary treatment for acute ischemic stroke with large vessel occlusion (AIS-LVO), providing substantial benefits, especially within the initial 6-h therapeutic window (Wahlgren et al., 2016; Bhan et al., 2020). However, a significant proportion of AIS-LVO patients, estimated to be approximately 30–40%, present to care facilities beyond this traditional window, specifically between 6 and 24 h from symptom onset (Jadhav et al., 2018; Gunda et al., 2021). While the DEFUSE 3 (Endovascular therapy following imaging evaluation for ischemic stroke 3) and DAWN (Diffusion-weighted imaging or computerized tomography perfusion assessment with clinical mismatch in the triage of wake up and late presenting strokes undergoing neurointervention with Trevo) trials have indicated the potential benefits of MT in this extended time window, they also highlighted the variability in outcomes (Jabal et al., 2023; Zhan et al., 2023), with beneficial functional outcomes at 90 days observed in roughly 45 and 49% of patients, respectively (Albers et al., 2018; Nogueira et al., 2018). This variation in outcomes suggests that successful reperfusion does not guarantee favorable recovery for a significant proportion of patients undergoing MT (Campbell et al., 2015). Hence, it necessitates the development of precise and time-efficient risk assessment tools to optimize patient selection, hence enhancing outcomes of those most likely to benefit from extended-window MT.

The intricate clinical and imaging biomarkers, along with their indirect, combined, or complex effects, present a significant challenge for traditional prediction models, such as logistic regression (Logit), which often struggle to capture the non-linear relationships between diverse prognostic factors (Drozdowska et al., 2019). Machine learning (ML) has emerged as a promising tool to handle high dimensional data and identify complex interactions among variables, and it holds great potential in optimizing outcome prediction models (Obermeyer and Emanuel, 2016). In particular, ML models that integrate various types of clinical and imaging features have shown potential in providing immediate prognostic information in time-sensitive situations such as acute stroke, thus supporting critical decision-making processes (Xie et al., 2019; Brugnara et al., 2020; Jiang et al., 2021).

However, research into utilizing ML for predicting the outcomes of extended-window MT in AIS-LVO patients remains limited. These complex clinical scenarios, with numerous interrelated clinical and imaging biomarkers, pose a significant challenge for the existing predictive models (Nishi et al., 2019). Additionally, the “black box” nature of many advanced ML models raises interpretability issues, leading to a trust gap among clinicians (Adadi and Berrada, 2018; Tjoa and Guan, 2021; Nazir et al., 2023). This lack of transparency, coupled with the need for research targeting this important clinical area, highlights the urgent need for predictive tools that are both applicable and interpretable in this specific scenario.

In response to the urgent need for interpretable predictive tools, this study proposes the introduction of an interpretation stage to the ML framework to enhance transparency and clinician confidence. Specifically, the utilization of Shapley additive explanation (SHAP), an approach offering solutions to the “black box” issue by elucidating each variable’s contribution to the prediction outcome, is at the core of our research. This study aims to develop a SHAP-interpreted ML model to predict the outcomes of extended-window MT in AIS-LVO patients. By integrating a diverse range of demographic, clinical, and neuroimaging variables, we plan to provide valuable insights into patient selection prior to MT, thereby contributing to more targeted treatment strategies and better clinical outcomes.

## Materials and methods

### Patient information

A retrospective analysis was conducted on a database of 260 AIS-LVO patients who were treated with MT and successfully reperfused at our center from January 2019 to January 2023. Included were patients (1) aged 18–90 years; (2) with confirmed occlusions in the anterior circulation, particularly in the M1/M2 segment of the middle cerebral artery (MCA) or intracranial internal carotid artery (ICA); (3) arrived at the emergency department 6–24 h after the last known well time or symptom onset, outside the standard 6-h therapeutic window; and (4) meeting the DEFUSE 3 trial eligibility criteria (Nogueira et al., 2018), namely an ischemic core (IC) volume < 70 mL, a mismatch ratio (MMR) ≥ 1.8, and a mismatch volume > 15 mL. The exclusion criteria were (1) patients with occlusions in the anterior cerebral artery or vessels with an internal diameter of less than 2 mm; (2) those with a premorbid modified Rankin Scale (mRS) score over 2; (3) patients with a history of intracranial hemorrhage, brain surgery, or significant territorial lesion; and (4) patients with any missing relevant clinical or radiological data. This study complied with the Helsinki Declaration and received approval from the institutional review boards of Shanghai Putuo Liqun Hospital (RT202204). Due to the retrospective nature of the investigation, the requirement for informed consent was waived. All data involved in this research were anonymized to uphold patient privacy.

### Pretreatment variables extraction

A wide range of pretreatment variables was extracted, including demographic, clinical, and neuroimaging data. The demographic and clinical variables comprised age, gender, onset-to-door time, and baseline national institutes of health stroke scale (NIHSS) score. Additionally, relevant comorbidities, such as hypertension, diabetes mellitus, hyperlipidemia, previous ischemic stroke, coronary heart disease, arterial fibrillation, and current smoking status, were incorporated into the clinical dataset.

Baseline neuroimaging data were obtained using a 64-slice multidetector CT scanner (Brilliance iCT; Philips Medical Systems, Best, Netherlands), which incorporated non-contrast CT (NCCT), CT angiography (CTA), and CT perfusion (CTP) scans. The neuroimaging variables consisted of the occlusion site, the Alberta stroke program early CT score (ASPECTS), and collateral scores.

CTP analysis was conducted utilizing the brain CT perfusion software, under the Philips IntelliSpace platform (Version 9.0, Brain CT Perfusion Package, Philips Healthcare, Best, Netherlands). By manually setting regions of interest corresponding to an artery and a vein, perfusion parameters including mean transit time (MTT), cerebral blood volume (CBV), and cerebral blood flow (CBF) were subsequently derived. Criteria for defining ischemic penumbra (IP) included a relative MTT > 150% and a CBV > 2.0 mL/100 g. Conversely, IC was characterized by a relative MTT > 150%, coupled with a CBV < 2.0 mL/100 g (Wintermark et al., 2006). The MMR was calculated by dividing the total hypoperfused tissue volume (the sum of the IC and IP volumes) by the IC volume.

### Data pre-processing

In preparation for an unbiased ML analysis, an essential step undertaken was the standardization of all variables, with the precise methodology varying based on the nature of the variable. Continuous variables were standardized to a scale with a mean of zero and a standard deviation of one. This standardization was crucial for continuous variables as it allowed them to contribute equally to the ML model, thereby enhancing its predictive performance, irrespective of their original scales (Ali et al., 2014). Categorical variables, on the other hand, were binarized and assigned a value of either “0” or “1.” Ordinal variables, such as ASPECTS and NIHSS scores, were scaled to lie within a [0, 1] range. An important component of the prognostic estimation was the dichotomization of the mRS score at 90 days (mRS-90), with scores ranging from 0 to 2 indicative of favorable outcomes (Saver et al., 2016). To ensure the robustness of the predictive model, the dataset was partitioned randomly into training and test subsets, following a 3:1 distribution.

### ML model derivation and validation

To predict favorable outcomes in AIS-LVO patients, four supervised ML classifiers—k-nearest neighbors (KNN), random forests (RF), support vector machine (SVM), and extreme gradient boosting (XGBoost)—were utilized. A 10-fold cross-validation strategy, combined with grid search algorithm, optimized the model hyperparameters and mitigated overfitting. The training set was partitioned into inner training and test subsets, rotating roles in subsequent iterations for robust validation. This fine-tuning was integral to achieving high model generalizability and accuracy. All algorithms, cross-validation procedures, and hyperparameter optimizations were implemented using the Python Scikit-Learn library.

A traditional Logit model was also developed for comparing the predictive capabilities of the ML models. Variables potentially correlated with favorable outcomes were evaluated using univariate Logit model. Subsequently, variables identified as significant in the univariate analysis (*p* < 0.05) were incorporated into the construction of the multivariate Logit model for outcome prediction.

After the model derivation, each model was subjected to a validation process to assess its discrimination, calibration, and clinical utility. Our selection for the optimal predictive model was guided by superior performance in discrimination, coupled with satisfactory results in both calibration and clinical utility.

### Model interpretability and testing

Following validation, the optimal predictive model was identified. We integrated the SHAP methodology for a more insightful interpretation of the model’s performance. Grounded in cooperative game theory, SHAP serves as a model-agnostic tool capable of elucidating predictions across various ML models (Chalkiadakis et al., 2012). It quantifies the average marginal contribution of each input parameter to a model’s prediction, providing a robust mechanism for evaluating feature importance (Martini et al., 2021). In our study, this method facilitated the calculation of absolute mean SHAP values for each feature by taking the mean of the absolute values of the SHAP values across all instances in the training set. This calculation disregards the direction of impact (positive or negative), focusing solely on the magnitude of influence each feature has on the model’s predictions, thereby enabling a rank ordering of feature importance. This process was crucial for understanding the specific contribution of each feature to the prediction, effectively identifying the most influential variables. It would significantly elevate the interpretability of our model, illuminating the pivotal predictors and their respective roles in the predictive outcomes observed within our training cohort of patients.

Further, the effectiveness of the model was rigorously evaluated using a test set. This assessment further affirmed its capabilities in terms of discrimination, calibration, and clinical utility, offering a more comprehensive understanding of its predictive capability.

### Statistical analysis

Statistical evaluations were conducted using the chi-square test or Fisher’s exact test for categorical variables, and the Mann–Whitney U test for ordinal variables. For continuous variables, the suitability of their distributions was evaluated via the Shapiro–Wilk test, guiding the use of either Mann–Whitney U test or independent-sample t-test accordingly. Model performance was evaluated with the receiver operating characteristic (ROC) curve, with the area under the curve (AUC) serving as a measure of model discrimination. Delong’s test facilitated comparisons among AUCs. Calibration curve analysis assessed the goodness of fit for each model. Moreover, decision curve analysis (DCA) was employed to estimate the net benefits associated with each model at varying threshold probabilities, providing insights into the clinical utility of the models. A two-tailed value of p of less than 0.05 was indicated statistical significance. Statistical processing of data was executed using IBM SPSS Statistics (v 22.0, SPSS Inc.) and Python (v 3.7.1).

## Results

### Patient characteristics

**Figure 1:**
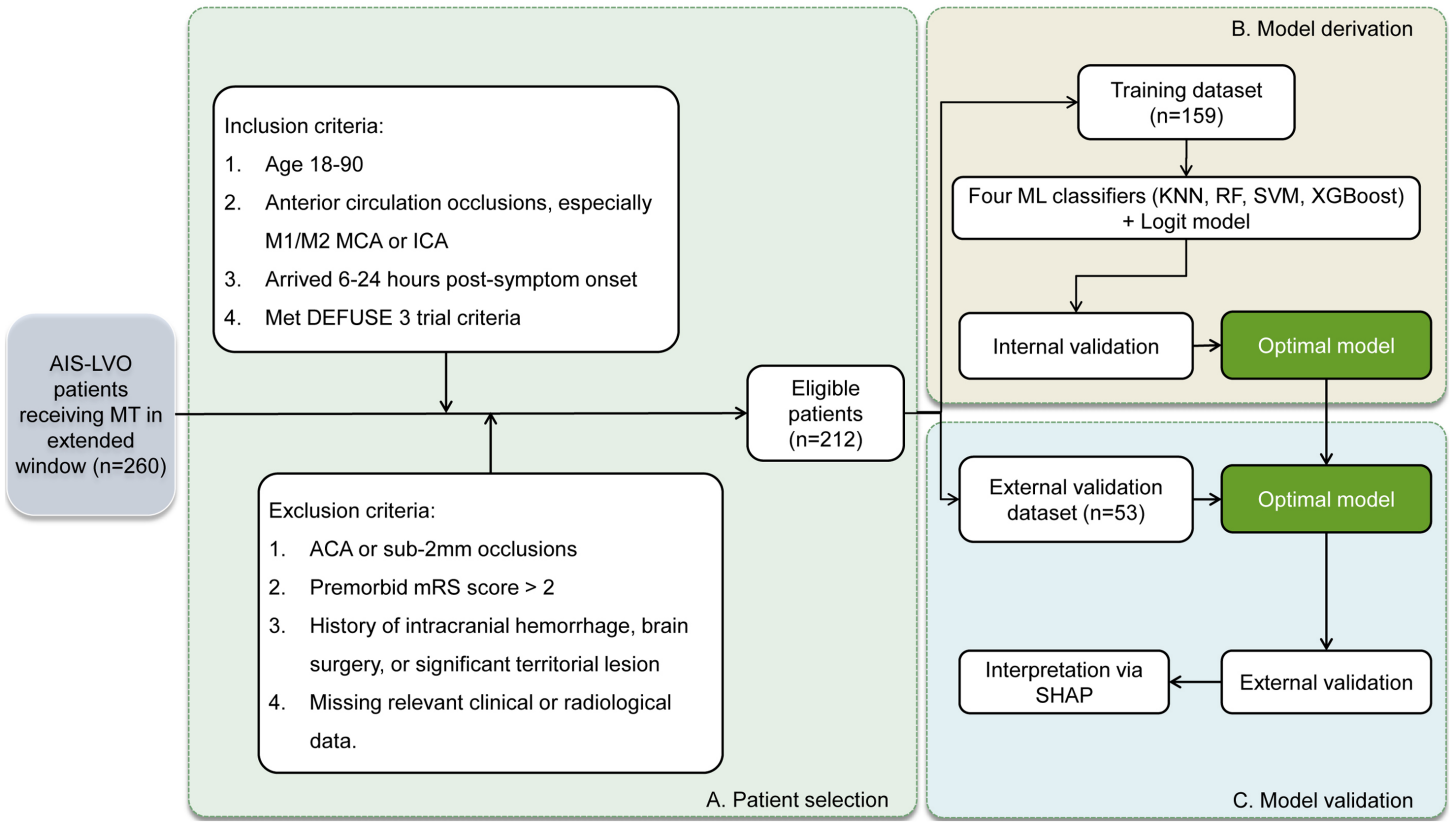
Workflow showing patient selection and model derivation and validation. AIS-LVO, acute ischemic stroke with large vessel occlusion; MT, mechanical thrombectomy; MCA, middle cerebral artery; ICA, internal carotid artery; DEFUSE 3, Endovascular therapy following imaging evaluation for ischemic stroke; ACA, anterior cerebral artery; mRS, modified Rankin Scale; ML, machine learning; KNN, k-nearest neighbors; RF, random forest; SVM, support vector machine; XGBoost, extreme gradient boosting; Logit, logistic regression; SHAP, Shapley additive explanation.

Figure 1 presents the flowchart of the patient selection process and model derivation and validation. From the initial pool of 260 patients, 212 (consisting of 126 males, mean age 68.2 ± 10.5 years) were selected for further analysis. The median baseline NIHSS score among these patients was 14, with an interquartile range (IQR) from 10 to 18. Distribution of occlusion sites was as follows: intracranial ICA (*n* = 25), MCA M1 segment (*n* = 153), and MCA M2 segment (*n* = 34). This cohort was divided into training (159 individuals) and test (53 individuals) datasets. Over a subsequent 90-day period, favorable functional outcomes were observed in 40.8% (65/159) of the training set and 37.7% (20/53) of the test set. Table 1 provides a comprehensive comparison of patient characteristics between these two datasets, indicating no significant differences in all evaluated parameters (all *p* > 0.05).

**Table 1:** Comparative analysis of demographic, clinical, and neuroimaging variables between the training and test sets.

Variables		Training set (*n* = 159)	Test set (*n* = 53)	*P*-value
Age, years		68.0 ± 10.6	68.9 ± 10.5	0.589^a^
Male, n(%)		93 (58.5%)	33 (62.3%)	0.628^c^
Baseline NHISS		13 (10, 17)	14 (12, 18)	0.163^b^
Onset-to-door time, h		10 (7, 13)	10 (7, 14)	0.583^b^
Comorbidities				
Diabetes, n(%)		42 (26.4%)	15 (28.3%)	0.788^c^
Hypertension, n(%)		80 (50.3%)	27 (50.9%)	0.937^c^
Hyperlipidemia, n(%)		27 (17.0%)	12 (22.6%)	0.357^c^
Previous ischemic stroke, n(%)		11 (6.9%)	3 (5.7%)	1.000^d^
Coronary heart disease, n(%)		26 (16.4%)	6 (11.3%)	0.376^c^
Arterial fibrillation, n(%)		48 (30.2%)	14 (26.4%)	0.601^c^
Smoking, n(%)		53 (33.3%)	14 (26.4%)	0.348^c^
Neuroimaging parameters				
ASPECTS		6 (5, 7)	6 (5, 7)	0.420^b^
Collateral score, n(%)	0	9 (5.7%)	4 (7.5%)	0.577^e^
	1	62 (39.0%)	23 (43.4%)	
	2	61 (38.4%)	21 (39.6%)	
	3	27 (17.0%)	5 (9.4%)	
IC volume, mL		10.8 (6.0, 17.4)	11.6 (7.4, 18.3)	0.475^b^
IP volume, mL		120.4 ± 47.6	115.8 ± 44.2	0.536^a^
MMR		11.5 (8.0, 21.1)	9.4 (7.3, 21.8)	0.334^b^
mRS-90 0–2 score, n(%)		65 (40.8%)	20 (37.7%)	0.686^c^

^a^ and ^b^ for numerical variables analyzed with independent-sample *t*-test and Mann–Whitney U test, respectively; ^c^ and ^d^ for categorical variables analyzed with chi-square test and Fisher’s exact test, respectively; ^e^ for ordinal variable analyzed with Mann–Whitney U test. NHISS, national institutes of health stroke scale; ASPECTS, Alberta stroke program early CT score; IC, ischemic core; IP, ischemic penumbra; MMR, mismatch ratio; mRS, modified Rankin Scale.

### Outcome-based comparison in the training set

Table 2 offers a comparison of various characteristics within the training set patients, categorized by their mRS-90 scores. The findings indicated that patients with favorable outcomes were typically younger, displayed lower baseline NIHSS scores, and experienced shorter onset-to-door times. Additionally, these patients showed distinct neuroimaging patterns, with reduced IC and IP volumes, elevated ASPECTS, and increased MMR. Moreover, a higher proportion of these patients had a collateral score of 2–3.

**Table 2:** Comparison of clinical, demographic, and neuroimaging characteristics stratified by mRS-90 scores in the training set.

Variables		Favorable outcome (*n* = 65)	Poor outcome (*n* = 94)	*P-*value
Age, years		62.9 ± 9.3	71.5 ± 10.1	<0.001^a^
Male, n(%)		34 (52.3%)	59 (62.8%)	0.188^c^
Baseline NHISS		10 (9,12)	16 (13, 19)	<0.001^b^
Onset-to-door time, h		8 (7, 11.5)	10 (7, 13)	0.011^b^
Comorbidities				
Diabetes, n(%)		14 (21.5%)	28 (29.8%)	0.246^c^
Hypertension, n(%)		28 (43.1%)	52 (55.3%)	0.129^c^
Hyperlipidemia, n(%)		10 (15.4%)	17 (18.1%)	0.656^c^
Previous ischemic stroke, n(%)		4 (6.2%)	7 (7.4%)	1.000^d^
Coronary heart disease, n(%)		9 (13.8%)	17 (18.1%)	0.477^c^
Arterial fibrillation, n(%)		18 (27.7%)	30 (31.9%)	0.569^c^
Smoking, n(%)		19 (29.2%)	34 (36.2%)	0.361^c^
Neuroimaging parameters				
ASPECTS		7 (6, 8)	6 (5, 7)	<0.001^b^
Collateral score, n(%)	0	0 (0.0%)	9 (9.6%)	0.004^e^
	1	22 (33.8%)	40 (42.6%)	
	2	26 (40.0%)	35 (37.2%)	
	3	17 (26.2%)	10 (10.6%)	
IC volume, mL		5.3 (3.5, 9.9)	16.0 (10.7, 21.1)	<0.001^b^
IP volume, mL		94.9 ± 33.0	137.9 ± 48.2	<0.001^a^
MMR		14.4 (8.8, 27.2)	10.1 (7.4, 16.5)	0.005^b^

^a^ and ^b^ for numerical variables analyzed with independent-sample *t*-test and Mann–Whitney U test, respectively; ^c^ and ^d^ for categorical variables analyzed with chi-square test and Fisher’s exact test, respectively; ^e^ for ordinal variable analyzed with Mann–Whitney U test. NHISS, national institutes of health stroke scale; ASPECTS, Alberta stroke program early CT score; IC, ischemic core; IP, ischemic penumbra; MMR, mismatch ratio.

### Model comparison for favorable outcomes

**Figure 2:**
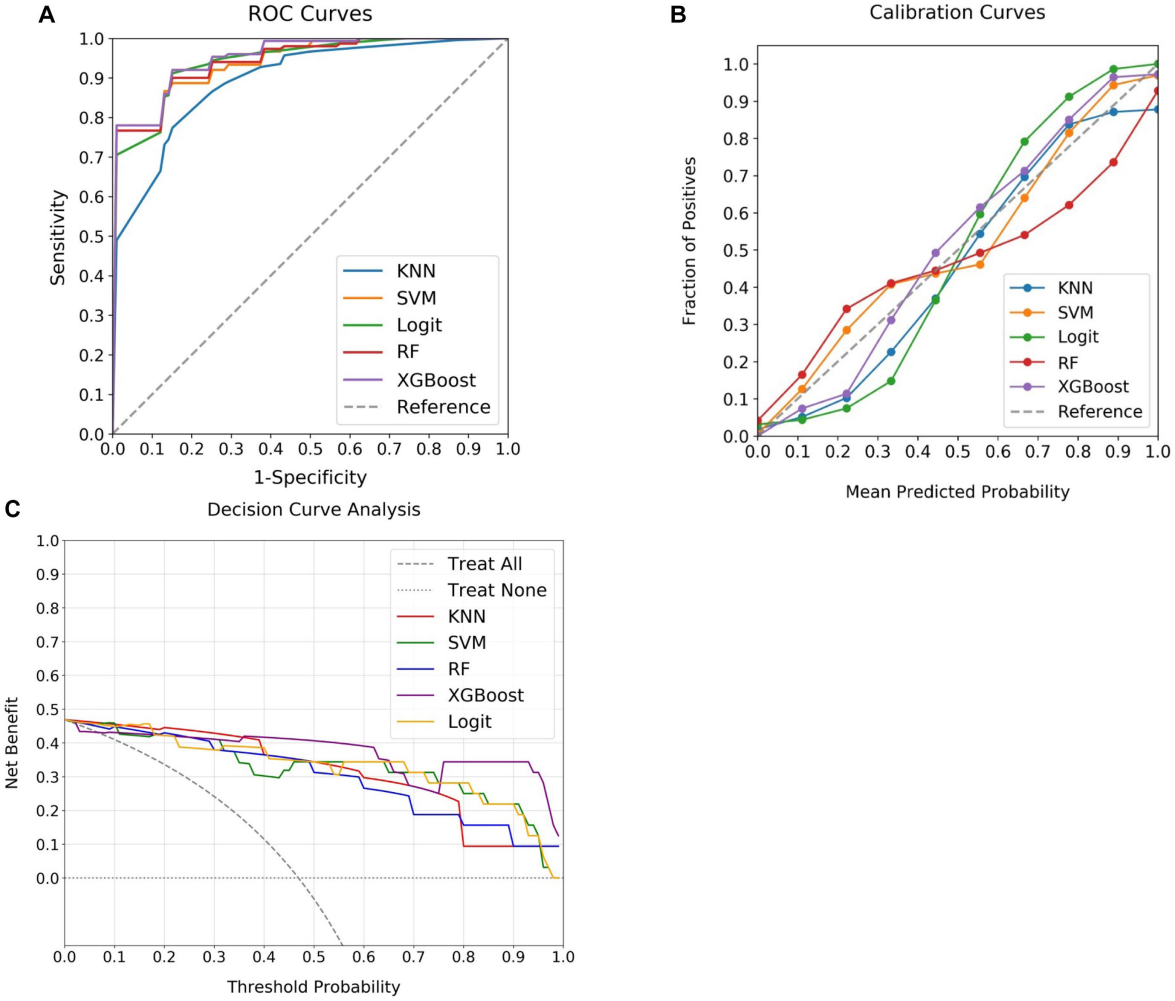
Comparative performance of ML classifiers and Logit model in predicting MT outcomes. **(A)** Presents the ROC curves, with XGBoost achieving the highest AUC (0.93), followed by SVM and RF (both 0.92), and Logit (0.89), while KNN reveals the lowest (0.86). **(B)** Illustrates calibration curves, where the X-axis shows the predicted probabilities and the Y-axis denotes the actual event frequency, with an ideal model aligning with the 45-degree line, signifying a precise match between predictions and observed outcomes. **(C)** Exhibits DCA, with the X-axis for threshold probabilities and the Y-axis for net benefit. “Treat All” represents a scenario of treating all individuals, while “Treat None” illustrates treating no individuals. The DCA reflects the net benefit of employing the models at various threshold probabilities compared to treating all or none. Despite minor variations, all models exhibit satisfactory performance in both calibration and DCA curves. ML, machine learning; MT, mechanical thrombectomy; KNN, k-nearest neighbors; SVM, support vector machine; Logit, logistic regression; RF, random forest; XGBoost, extreme gradient boosting; ROC, receiver operating characteristic; DCA, decision curve analysis.

In the assessment of MT outcomes, a comparative analysis was conducted employing four ML classifiers (KNN, SVM, RF, and XGBoost) alongside a Logit model, with findings illustrated in Figure 2. The ROC curves (Figure 2A) delineated significant variations among the models, where XGBoost exhibited the highest discriminatory power with an AUC of 0.93, while SVM, RF, Logit, and KNN followed with AUCs of 0.92, 0.92, 0.89, and 0.86 respectively; however, the difference between XGBoost and KNN was not statistically significant (*p* > 0.05, DeLong test). Moving on to the calibration curves (Figure 2B), minor deviations were noted, with Logit and RF models showing slightly lower alignment, yet all models demonstrated reliable performances. In the DCA (Figure 2C), XGBoost displayed superior efficacy, while RF indicated somewhat lower performance. Therefore, XGBoost emerged as the optimal model, exhibiting exemplary predictive performance with a Precision of 0.93, a Recall of 0.87, and an F1 Score of 0.90, marking it as the best among those analyzed.

### Testing of the optimal model

**Figure 3:**
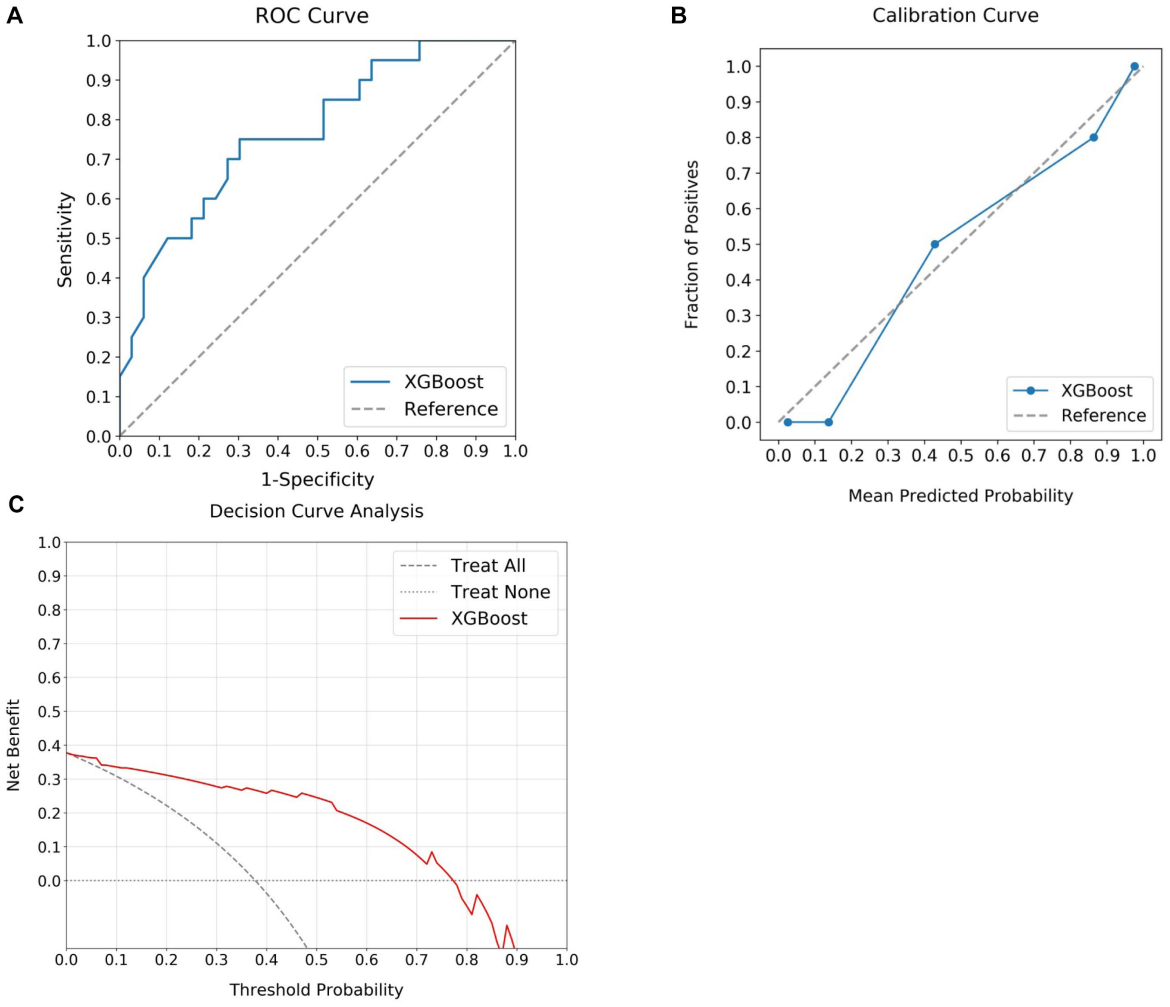
Assessment of MT outcomes using the optimal predictive model in the test set. **(A)** Illustrates the discriminative capability of the model with a notable AUC of 0.77. **(B)** Presents the calibration curve, demonstrating a strong agreement between the predicted and actual outcome. **(C)** Shows the DCA curve, emphasizing the substantial net benefits delivered by the model over a prediction probability range from 0 to 0.8. MT, mechanical thrombectomy; XGBoost, extreme gradient boosting; ROC, receiver operating characteristic; AUC, area under the curve; DCA, decision curve analysis.

The XGBoost model, identified as the optimal predictive model, was subjected to further evaluation with a test dataset. Variables from this set were applied to the XGBoost model, followed by a comparison between the predictions and actual patient outcomes. This analysis is illustrated through the ROC, calibration, and DCA curves in Figure 3. Despite a slight reduction in performance relative to the training set, the XGBoost model demonstrated considerable discriminative power, with an AUC of 0.77 on the ROC curve (Figure 3A). The calibration curve revealed a strong alignment between the predicted probabilities and actual event frequencies (Figure 3B). The DCA curve further displayed significant net benefits across prediction probabilities ranging from 0 to 0.8 (Figure 3C), further establishing the potential of the XGBoost model in predicting MT outcomes. Additionally, the model manifested a Precision of 0.90, a Recall of 0.70, and an F1 Score of 0.79, indicating satisfactory predictive capacity.

### Model interpretation

**Figure 4:**
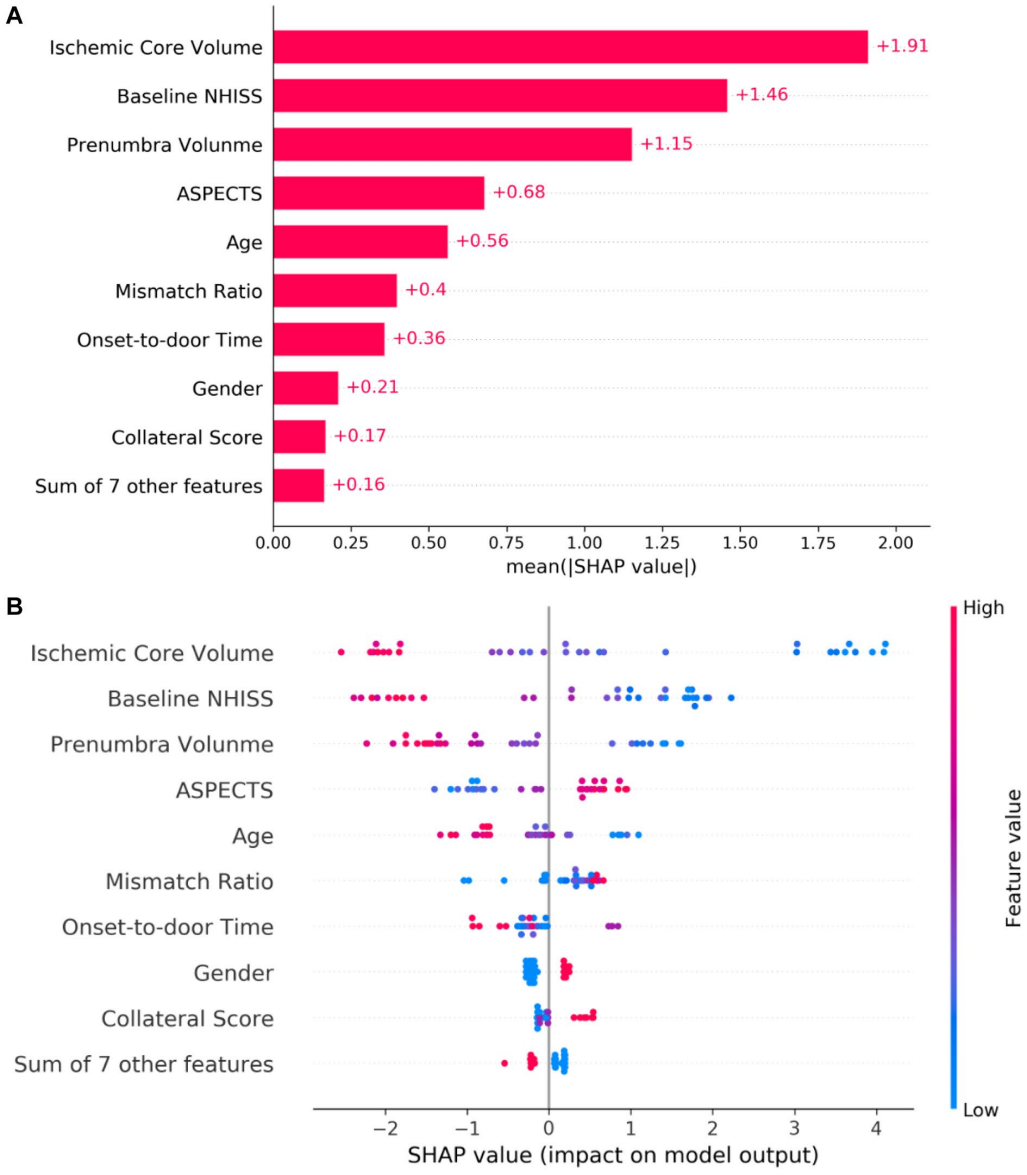
Interpretation of the predictive model via SHAP Analysis. In **(A)**, the absolute mean SHA*p* values demonstrate the global impact of each feature on the model prediction. Features are ranked along the y-axis based on their importance, with those at the top contributing more to the model. **(B)** Presents a summary of SHAp values for each feature, illustrating the relationship between the feature value and its effect on the model prediction. Each dot corresponds to an individual patient. The color gradation from blue to red reflects the value of the feature, with redder dots indicating higher values and bluer dots indicating lower values. The horizontal axis denotes the SHAP value corresponding to each feature. Positive SHAP values contribute positively to the MT outcome prediction and vice versa. The position of a dot along the x-axis indicates the degree of impact that the corresponding feature has on the model prediction for a specific patient. SHAP, Shapley additive explanation; NHISS, national institutes of health stroke scale; ASPECTS, Alberta stroke program early CT score; MT, mechanical thrombectomy.

SHAP analysis, which quantifies the influence of individual features within the ML model, was utilized for interpreting the XGBoost model. IC volume, baseline NIHSS score, IP volume, ASPECTS, and patient age emerged as the top five key determinants (Figure 4A). To visually represent the cumulative impact of each variable, a summary plot of the SHAP values was constructed (Figure 4B). This plot enables a detailed understanding of how each predictor influences predictions for individual patients. The analysis demonstrated that the model associated smaller IC volume, lower NIHSS score, reduced IP volume, higher ASPECTS, and younger age with an increased likelihood of achieving a favorable outcome after MT.

## Discussion

An accurate preoperative evaluation of MT prognosis is crucial, considering that successful reperfusion does not necessarily correspond to a favorable recovery. This is particularly true for patients in the extended therapeutic window, as they might exhibit more complex clinical outcomes owing to prolonged ischemic time. In the present study, we conducted a comparative analysis of the prognostic capabilities of four ML classifiers and a Logit model, based solely on clinical and imaging features readily available in the emergency department. Our findings indicated that the XGBoost model outperformed others in terms of optimal discrimination, satisfactory calibration, and clinical utility in both the training and test datasets. Notably, the application of SHAP enhanced the interpretability and transparency of the XGBoost model, illuminating the fundamental features influencing stroke outcomes. By leveraging such knowledge, it might be feasible for clinicians to individualize treatment plans for optimizing clinical outcomes, and offer timely and personalized care driven by the results of the ML model, thereby potentially enhancing management in AIS-LVO patients.

In our study, we compared the prognostic performance of four ML models—KNN, SVM, RF, and XGBoost—against the conventional Logit model. These ML models are particularly suited to handle intricate non-linear relationships between variables and outcomes, giving them an edge over Logit (Uddin et al., 2019; Silva et al., 2022). While all models showed similar calibration and clinical utility, their performance varied substantially in terms of discrimination. KNN, being sensitive to noise and outlier data points, along with its degraded performance in complex and high-dimensional datasets (Abu Alfeilat et al., 2019), showed the lowest discrimination. On the other hand, SVM, RF, and XGBoost, with their superior ability to manage non-linear relationships, outperformed Logit. Consistent with previous research (Heo et al., 2019; Chiu et al., 2021; Zhang et al., 2022), our study reinforced the superiority of ML models over Logit predicting outcomes of endovascular treatment for stroke. However, most existing studies are confined to a 6-h therapeutic window, with limited research focusing on ML predictions for extended-window MT. A study by Lu et al. (2022) remains the exception, although it primarily builds predictive models based on variables available post-MT, providing minimal insights into MT decision-making. Given the sizable cohort of AIS patients presenting beyond the therapeutic window, it becomes crucial to rapidly and precisely predict the potential benefits of MT, especially within the emergency department setting. To address this need, our models strategically incorporated only pre-intervention clinical and imaging variables. These purposefully designed predictive models could assist in identifying patients most likely to benefit from MT, potentially enhancing both the decision-making process and therapeutic outcomes for these patients.

This study represented the first application of ML models to predict clinical outcomes in AIS-LVO patients undergoing MT within the extended therapeutic window. To address the interpretability challenges intrinsic to complex ML models, the study incorporated the SHAP methodology. This approach offers a transparent illustration of decision-making processes at the cohort level, augmented by user-friendly visualization tools (Nohara et al., 2022). The feature importance offered by SHAP elucidates the contribution of individual variables to the model’s predictive power, facilitating trust between clinicians and AI algorithms (Alabi et al., 2022; Jabal et al., 2022; Xiong et al., 2022). Further investigation of the XGBoost model, validated as the optimal ML model for predicting MT outcomes, revealed key predictors including IC volume, baseline NIHSS score, IP volume, ASPECTS, and patient age.

While baseline NIHSS score, ASPECTS, and patient age are well-established predictors in stroke prognosis, the enhanced predictive accuracy of the present model was primarily attributed to the inclusion of IC and IP volumes. This enhancement finds support in the collective findings of O'Connor et al. (2020), Hamann et al. (2021), and Zhang et al. (2022), who all emphasized the significance of cerebral infarction volume and CTP-derived core volumes in ML models for predicting MT outcomes. However, a common limitation across these studies was their focus on patients within the standard therapeutic window. In research extending beyond this window, Lu et al. (2022) also identified IC volume and mismatch volume as key variables in their ML models, yet the predictive accuracy was compromised, likely due to a limited sample size.

These variables delivered enhanced performance within the XGBoost framework. The robustness of the XGBoost model can be attributed to its proficiency in handling and interpreting complex non-linear relationships between variables and outcomes (Sheridan et al., 2016). As a gradient boosting algorithm, XGBoost captures sophisticated, non-linear relationships through iterative construction and optimization of decision trees, thereby unmasking intricate data patterns (Torlay et al., 2017; Mateo et al., 2021). In conjunction with SHAP, XGBoost allows for a transparent depiction of the significant influence each variable has on the predicted outcome. This unique combination equips XGBoost as an efficient tool in the rapid identification of AIS patients who are likely to benefit from MT, even beyond the standard therapeutic window, within the high-pressure environment of an emergency department. Notably, its reliance on readily available pre-intervention clinical and imaging variables eliminates the need for additional testing technologies, thereby preventing any increase in clinical burden. This efficient integration of ML in the decision-making process highlights its potential to revolutionize stroke management, and ultimately improve patient outcomes.

In acknowledging the limitations of this study, it is first essential to consider that our research was conducted in a single institution, using a specific CT scanner, and on a relatively limited patient cohort. These factors could introduce inter-observer variability due to potential differences in equipment or operators, which could, in turn, impact the efficacy of the employed ML models. Second, the retrospective design of the study and adherence to the DEFUSE 3 trial selection criteria might limit the wider applicability of our findings across different patient populations and therapeutic settings. Lastly, to maintain clinical feasibility, the ML model incorporated only standard pre-intervention variables. Although the inclusion of novel techniques or indicators may enhance the discriminative power of the model, such improvements necessitate further rigorous validation. Future investigations should be planned to conduct larger, multi-center, prospective studies to further improve the reliability and precision of the model. The introduction of inter-rater reliability tests could help offset potential observer variability, thereby enhancing the reliability and widespread applicability of the model.

## Conclusion

This study represented an important progress in the management of AIS-LVO patients through the development of an interpretable ML model. By incorporating routinely available clinical and imaging variables, this model held the potential to accurately identify patients suitable for MT within an extended therapeutic window. The incorporation of SHAP analysis not only strengthened the interpretability of the model but also promoted its reliability in clinical settings. By providing accurate predictions of three-month post-MT functional outcomes, this model had the potential to guide the development of personalized and effective treatment strategies, thereby paving the way for improved patient outcomes.

## Data availability statement

The raw data supporting the conclusions of this article will be made available by the authors, without undue reservation.

## Ethics statement

The studies involving humans were approved by the Institutional Review Boards of Shanghai Putuo Liqun Hospital (RT202204). The studies were conducted in accordance with the local legislation and institutional requirements. The participants provided their written informed consent to participate in this study.

## Author contributions

LT: Conceptualization, Formal analysis, Methodology, Software, Supervision, Validation, Visualization, Writing – original draft, Writing – review & editing. YS: Conceptualization, Data curation, Formal analysis, Software, Writing – original draft, Writing – review & editing. YZ: Data curation, Formal analysis, Software, Writing – review & editing. HL: Data curation, Formal analysis, Investigation, Writing – review & editing. WW: Data curation, Formal analysis, Software, Writing – review & editing. YW: Conceptualization, Funding acquisition, Investigation, Project administration, Resources, Supervision, Writing – original draft, Writing – review & editing.
